# Persistent northward North Atlantic tropical cyclone track migration over the past five centuries

**DOI:** 10.1038/srep37522

**Published:** 2016-11-23

**Authors:** Lisa M. Baldini, James U. L. Baldini, Jim N. McElwaine, Amy Benoit Frappier, Yemane Asmerom, Kam-biu Liu, Keith M. Prufer, Harriet E. Ridley, Victor Polyak, Douglas J. Kennett, Colin G. Macpherson, Valorie V. Aquino, Jaime Awe, Sebastian F. M. Breitenbach

**Affiliations:** 1 Department of Earth Sciences, Durham University, Durham, DH1 3LE, UK; 2 Department of Geosciences, Skidmore College, 815 North Broadway, Saratoga Springs, New York, 12866, USA; 3 Department of Earth and Planetary Sciences, University of New Mexico, Albuquerque, NM 87106, USA; 4 Department of Oceanography and Coastal Sciences, Louisiana State University, Baton Rouge, LA 70803, USA; 5 Department of Anthropology, University of New Mexico, Albuquerque, NM 87106, USA; 6 Department of Anthropology, The Pennsylvania State University, University Park, Pennsylvania 16802, USA; 7 Institute of Archaeology, Belmopan, Belize; 8 Department of Anthropology, Northern Arizona University, Flagstaff, AZ 86011-5200, USA; 9 Department of Earth Sciences, University of Cambridge, Downing Street, Cambridge, CB2 3EQ, UK; 10 Institute for Geology, Mineralogy & Geophysics, Ruhr-Universität Bochum, Unversitätsstr. 150, 44801, Bochum, Germany

## Abstract

Accurately predicting future tropical cyclone risk requires understanding the fundamental controls on tropical cyclone dynamics. Here we present an annually-resolved 450-year reconstruction of western Caribbean tropical cyclone activity developed using a new coupled carbon and oxygen isotope ratio technique in an exceptionally well-dated stalagmite from Belize. Western Caribbean tropical cyclone activity peaked at 1650 A.D., coincident with maximum Little Ice Age cooling, and decreased gradually until the end of the record in 1983. Considered with other reconstructions, the new record suggests that the mean track of Cape Verde tropical cyclones shifted gradually north-eastward from the western Caribbean toward the North American east coast over the last 450 years. Since ~1870 A.D., these shifts were largely driven by anthropogenic greenhouse gas and sulphate aerosol emissions. Our results strongly suggest that future emission scenarios will result in more frequent tropical cyclone impacts on the financial and population centres of the northeastern United States.

Observational and modelling studies suggest that the recent multidecadal trend of rising sea surface temperatures (SST) in the North Atlantic’s Main Development Region (MDR) may have increased Atlantic tropical cyclone (TC) intensity and duration^1^,^2^,^3^, and shifted storm tracks poleward^4^,^5^. Some studies ascribe this oceanic warming to a multi-decadal SST periodicity known as the Atlantic Multidecadal Oscillation (AMO)^6^,^7^ associated with the strength of thermohaline circulation^7^,^8^ or large-scale atmospheric circulation^9^,^10^, while others implicate rising anthropogenic greenhouse gases (GHGs)^11^,^12^. Deconvolving these effects is critical for predicting how GHG-induced 21^st^ Century warming may impact future TC activity^13^, however the observational record’s brevity complicates assessing the relative influence of natural versus anthropogenic climate forcings on past North Atlantic TC activity. Additionally, multi-model ensemble studies predict that overall TC frequency will decrease through the 21^st^ Century while the frequency and intensity of the largest storms will increase^14^. Although global TC activity and strength predictions are reasonably well constrained, projections for individual basins have considerably more uncertainty^15^. Consequently, understanding the drivers of TC strength, frequency, and track for individual basins is critical. Well-dated, high resolution proxy records of total TC activity (including weaker tropical storms) from multiple individual locations are required^16^ to identify and characterise long-term trends in North Atlantic TC activity and the dominant geographic distribution of TC tracks prior to the historical and satellite eras^4^,^17^.

Here, we use coupled monthly-resolved oxygen and carbon isotope ratio (δ^18^O and δ^13^C) data from a Belizean stalagmite to reconstruct western Caribbean TC activity since 1550 A.D. Stalagmite YOK-G was collected from Yok Balum Cave (16° 12′ 30.780″ N, 89° 4′ 24.420″ W; 336 m.a.s.l.) in southern Belize in 2006. The stalagmite chronology is extremely robust, constructed using well-defined annual δ^13^C cycles that were counted from 1550 to 1983 and verified against nineteen very high precision MC-ICP-MS ^230^Th dates^18^. To construct the YOK-G tropical cyclone activity (YOK-G_TC_) record, the component of δ^18^O_p_ affected by TC activity^19^ was identified by removing scaled YOK-G δ^13^C values (reflecting rainfall amount) from scaled YOK-G δ^18^O values (reflecting rainfall amount and δ^18^O_p_) (see Methods Section). The resulting composite record was calibrated against the HURDAT2^20^ western Caribbean TC count over the interval 1900–1983 yielding a correlation that was significant at the 99.8% confidence level (Fig. 1) (see Methods Section). The resulting YOK-G_TC_ reconstruction spans the period 1550 to 1983 (Fig. 1) and includes both Cape Verde (originating within the MDR west of Africa)^6^ and non-Cape Verde storms (originating nearby in the western Caribbean)^21^,^22^. BH strength and position exert a significant control on Cape Verde TC track positions, with straighter east-west trajectories commonly associated with a weaker BH (a negative NAO phase)^22^. Conversely, TCs originating in the western Caribbean generally track northwards and are unrelated to MDR SSTs or BH strength^22^.

## Results

**Figure 1:**
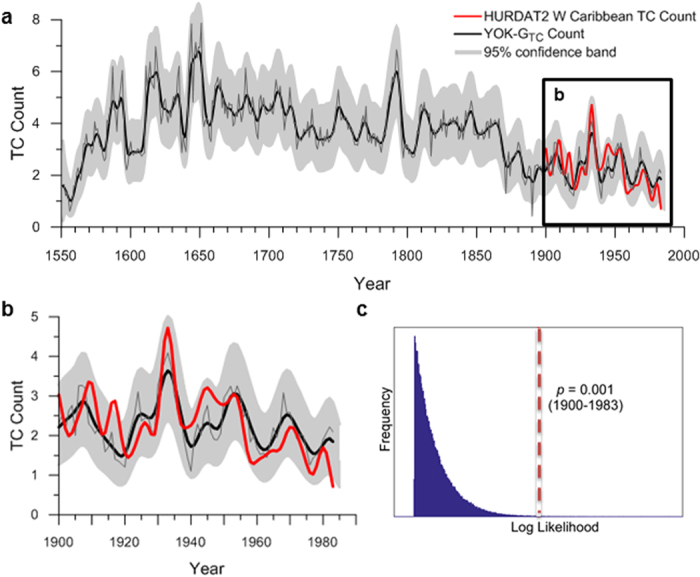
The YOK-G_TC_ reconstruction and observational record calibration. (**a**) The smoothed (black curve) and unsmoothed (grey curve) YOK-G_TC_ reconstruction back to 1550 A.D. (**b**) Modern calibration with the HURDAT2 western Caribbean TC count (red curve) from 1900 to 1983. Shading in a and b indicates the 95% confidence band. (**c**) Histogram of the log likelihood values versus frequency. The dashed red line represents the log likelihood (probability) that the actual data correlates with the HURDAT2 western Caribbean TC count by chance (*p* = 0.001).

**Figure 2:**
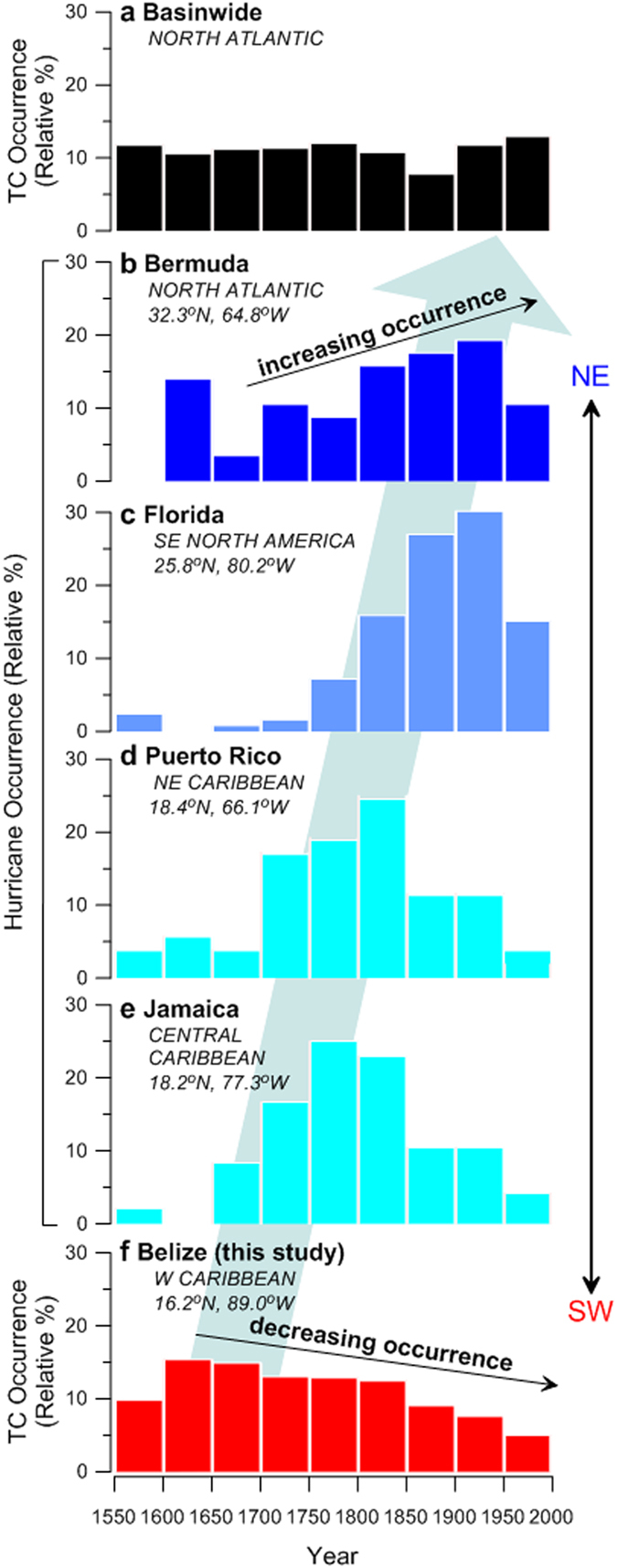
The YOK-G_TC_ reconstruction compared to documentary records of hurricane landfall in the Caribbean and North Atlantic Basins. Frequency distributions (relative %) of hurricanes affecting (**a**) the entire North Atlantic Basin and locations along the western margin of the North Atlantic (**b**) Bermuda, (**c**) Florida, (**d**) Puerto Rico, and (**e**) Jamaica, calculated from previously published documentary data^26^,^27^,^57^. Data are presented in 50-year time slices between 1551 and 1998, and are compared to the frequency distribution of TCs affecting (**f**) Belize (this study). The relative % occurrence for each site represents the total number of storms recorded during each 50-year time slice compared with the total number of storms that impacted the site since 1551 A.D. Because the YOK-G_TC_ record terminates at 1983, the final 1951 to 1998 time slice presented in (**f**) is based on the HURDAT2 western Caribbean TC count. The blue arrow illustrates the north-eastward progression of mean TC track schematically. The apparent decrease in relative % hurricane occurrence at all sites since 1950 is a result of numerous storms that passed within 320 km of Florida and Bermuda since 1950 but not close enough to affect the observational record (see Supplementary Information).

The YOK-G_TC_ reconstruction suggests that in the mid-16^th^ Century, on average, only one TC affected the western Caribbean region per year. This represents the lowest TC activity over the interval of our study (Fig. 1a), and is consistent with other regional reconstructions^23^,^24^,^25^ (see Supplementary Information). The YOK-G_TC_ count peaks at approximately eight storms per year during the 17^th^ Century (1σ = ±1.2) after which it decreases steadily until ~1870, when an abrupt decrease (from ~four to ~two storms annually) occurs, followed by muted TC frequency and variability (1σ = ±0.6) (Fig. 1a). In broad terms, this could reflect either a decrease in basin-wide activity or a repositioning of mean TC track away from the western Caribbean. No evidence exists for a secular basin-wide TC activity decrease since 1650 A.D.^26^ (Fig. 2a); our record combined with observational hurricane landfall records from Bermuda, Florida, Puerto Rico, and Jamaica^27^ instead support mean TC track migration to the northeast since 1650 A.D. (Fig. 2). This pattern of contrasting TC frequencies between Belize (Fig. 2f) and more northeasterly sites (Fig. 2b,c) is also consistent with an out-of-phase relationship inferred from lower resolution regional TC reconstructions^28^,^29^,^30^ (see Supplementary Information), and from satellite-based TC track studies during recent decades^17^.

**Figure 3:**
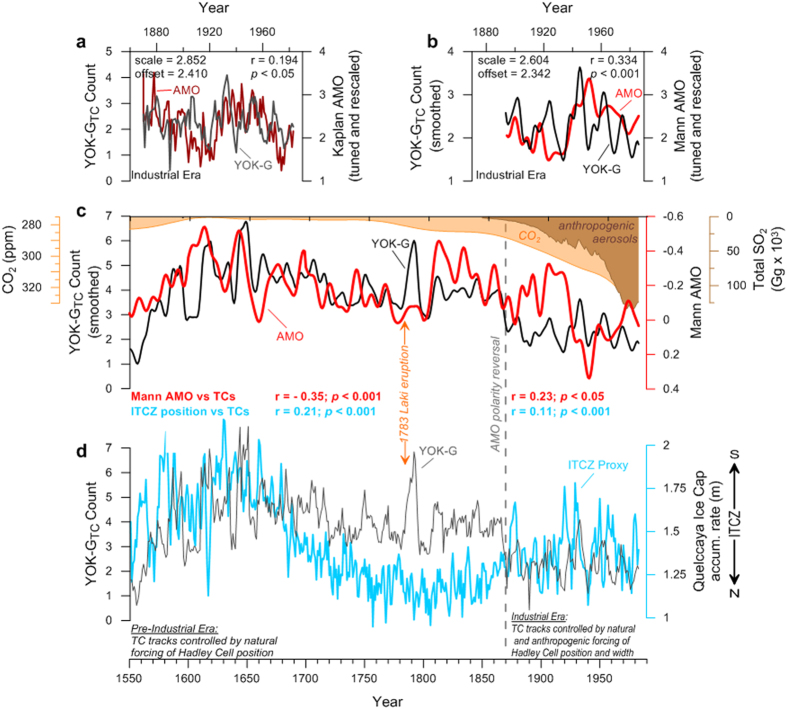
The YOK-G_TC_ reconstruction compared to two AMO indices and a proxy of ITCZ position. (**a**) The Kaplan SST AMO Index^35^ and (**b**) the Mann *et al*.^34^ AMO Index rescaled to have the same mean and standard deviation as the YOK-G_TC_ count and tuned within errors to reveal optimal fit parameters over the Instrumental interval. (**c**) The multi-decadally smoothed YOK-G_TC_ count (thick black curve) compared to the Mann *et al*. multi-decadally smoothed AMO reconstruction^34^ (red curve). Also shown are the 75-yr smoothed historical CO_2_ record from the Law Dome ice core^58^, Antarctica, (orange shaded curve) and total anthropogenic SO_2_ emissions since 1850^59^ (brown shaded curve). The approximate timing of the polarity reversal discussed in the text is represented by the dashed grey line at 1870. Note that the records are not tuned as in a and b, and that the axis for the AMO record is inverted to that in (**b**). (**d**) The annually resolved YOK-G_TC_ count (thin dark grey curve) compared to the 3-yr moving average of the Quelccaya Ice Cap (Peru) accumulation rate (in meters water equivalent per year) as a proxy for ITCZ position^38^. An increased ice accumulation rate occurs when the ITCZ is positioned southward over Peru. The results of linear least squares regression analysis are shown. Regression results in (**c**) and (**d**) are based on 5-yr moving averages of the datasets.

**Figure 4:**
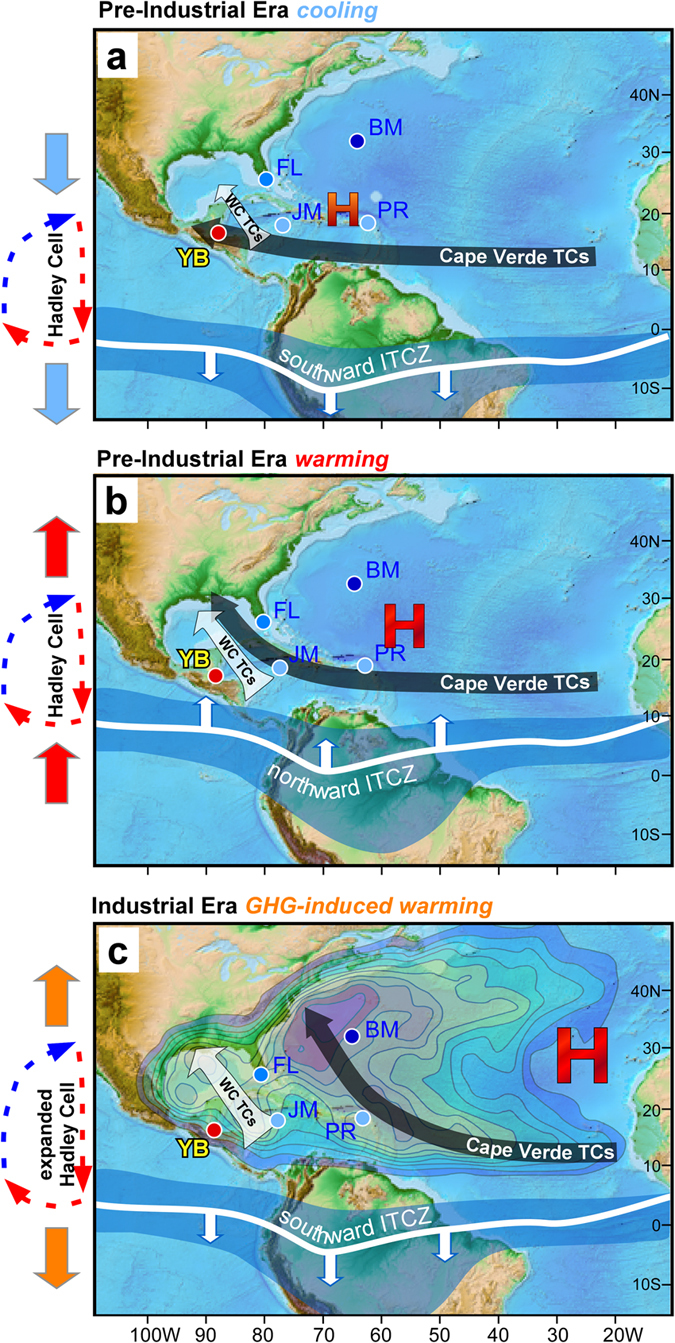
Generalised TC track migration patterns during the pre- and post-1870 intervals. Mean track of Cape Verde TCs (long black arrow) and the positions of the ITCZ (blue band) and the Bermuda High (red ‘H’) during (**a**) pre-Industrial LIA cooling, (**b**) pre-Industrial (post-LIA) warming, and (**c**) post-1870 GHG warming. Sites discussed in Fig. 3 are marked by circles (Yok Balum Cave (YB), Jamaica (JM), Puerto Rico (PR), Florida (FL), and Bermuda (BM)). Colour contours in (**c**) represent the likelihood a TC will occur during the Atlantic hurricane season (June 1–November 30) for the period 1944 to 1999 (adapted from ref. 60). The location and size of the red ‘H’s in a-c approximate BH position and strength, respectively. The position of the BH in (**c**) is based on 20^th^ Century Reanalysis V2 wind vector data^61^. ITCZ latitudinal position and shape is approximated from previous work^37^,^62^. The base map was derived from the ETOPO1 1 Arc-Minute Global Relief Model^63^. The size of the light grey arrows in a-c represents the relative importance of western Caribbean cyclogenesis during each interval schematically.

Previous research has suggested that the AMO is an important driver of North Atlantic TC activity^7^,^10^,^31^,^32^,^33^. YOK-G_TC_ and the AMO^34^,^35^ are in fact positively significantly correlated from 1870 to 1983 (Fig. 3a–c), but surprisingly are significantly anticorrelated before 1870 (Fig. 3c). The timing of the polarity shift in the YOK-G_TC_-AMO relationship at ~1870 A.D. is synchronous with the advent of widespread industrialisation and suggests an anthropogenic cause. We propose that this polarity reversal reflects the combined effects of GHGs and atmospheric aerosols on Hadley Cell width and position (Fig. 3). At ~1650 A.D. (within the range of peak LIA cooling) the ITCZ and NH Hadley Cell were at their southernmost extent^36^,^37^,^38^ (Figs 3 and 4). A southwesterly displaced BH (consistent with a strongly negative NAO^36^), steered Cape Verde TCs towards Central America and the Gulf Coast, resulting in the TC maximum evident in the YOK-G_TC_ record. The gradual YOK-G_TC_ activity decrease after 1650 A.D. is consistent with observational and modelling studies showing gradual northward ITCZ, Hadley Cell, and BH repositioning due to AMO warming (and increasing NH temperature) from peak LIA conditions^22^,^38^,^39^,^40^ (Figs 3 and 4). Following industrialisation, rising atmospheric GHG concentrations expanded the Hadley cells^41^,^42^,^43^ while rising anthropogenic sulphate aerosol emissions shifted the ITCZ southward^18^,^38^,^44^,^45^,^46^. An expanded NH Hadley Cell resulted in northward BH displacement despite a more southerly ITCZ, and consequently forced a northward migration of Cape Verde TCs (Fig. 4c) away from the western Caribbean. This effect is superimposed on a southward migration of the MDR, which tracks the southward migration of the ITCZ^5^. The abrupt western Caribbean TC decrease at ~1870 may reflect a shift to more northerly recurving tracks^47^ for one or two Cape Verde storms per year that had previously impacted the Yok Balum Cave site, a scenario supported by contemporaneous TC activity increases at more northeasterly sites such as Bermuda and Florida^27^ (Fig. 2). Although earlier industrialisation (i.e., from the late 18^th^ Century to 1870) undoubtedly also had an effect, our results suggest that the threshold where several storms no longer affected the western Caribbean was only passed at ~1870, implying that the average Cape Verde TC track moved north of our site at this time; the threshold at sites further to south may have been passed earlier in the industrial interval. Higher Caribbean SSTs^48^ promoting increased western Caribbean cyclogenesis resulted in the positive correlation between the YOK-G_TC_ count and the AMO post-1870. Our interpretations are also supported by a spike in the YOK-G_TC_ count occurring at 1783 A.D. (Fig. 3). The large influx of sulphate aerosols into the NH during the climatologically important Laki volcanic eruption may have cooled the NH resulting in fewer North Atlantic TCs overall^49^; however, our results suggest that the eruption also shifted North Atlantic TC tracks to the south, resulting in relatively more Central American TC landfalls.

## Discussion

The YOK-G_TC_ reconstruction strongly suggests that gradual warming since 1650 A.D., exacerbated by anthropogenic effects after 1870, forced a progressive decrease in western Caribbean TC activity while simultaneously increasing TC landfall frequency along the North American east coast. The YOK-G_TC_ record confirms the AMO as an important driver of western Caribbean TC activity, but reveals a polarity reversal in the relationship at ~1870, most likely due to GHG- and aerosol-induced changes in the teleconnection between the ITCZ and the BH across the pre-Industrial era and the Industrial Era transition. Our results suggest that although western Caribbean TC activity during the Industrial Era is within the pre-Industrial range, anthropogenic GHG and aerosol emissions have clearly repositioned mean TC tracks northward.

In the 21^st^ Century, atmospheric GHGs and Southern Hemisphere sulphate aerosol emissions are expected to continue rising while NH atmospheric aerosol emissions are projected to decrease^50^, resulting in increased potential^51^,^52^ and actual intensities^53^,^54^ of TCs along with an overall reduction in global TC frequency^12^,^14^,^15^. Under such conditions, our results suggest that Hadley Cell expansion (due to increasing GHG concentrations^41^) combined with northward ITCZ displacement (due to predicted reductions in NH aerosol emissions)^37^,^41^ will increasingly direct long-lived Cape Verde TCs further to the northeast. In the Caribbean, higher SSTs^55^ may promote western Caribbean cyclogenesis, replacing future losses of Cape Verde storms; consequently TC activity across this region may remain essentially stable over the current century. However, our results have important consequences for the global financial and population centres of the mid-Atlantic and New England regions of the USA, where policymakers should prepare for more frequent landfalls of more powerful TCs.

## Methods

### YOK-G δ^18^O

While the YOK-G δ^13^C record was previously interpreted as reflecting local rainfall amount^18^, the YOK-G δ^18^O record was largely uninterpreted until now. Although several variables such as moisture source, rainfall amount, temperature, and moisture mass trajectory influence precipitation δ^18^O (δ^18^O_p_), a dominant control on δ^18^O_p_ in tropical regions such as Belize is rainfall intensity (or ‘amount’). Tropical cyclone rainfall is characterised by particularly low δ^18^O_p_ values due to extensive fractionation of uplifted water vapour^19^ and this isotopic depletion can extend for several 100 km from the storm’s eye (see Supplementary Information); these low δ^18^O_p_ values are then transmitted to the growing stalagmite via drip water. To isolate the TC signal within the YOK-G isotope record, scaled YOK-G δ^13^C values (reflecting rainfall amount) were removed from scaled YOK-G δ^18^O values (reflecting the combined influence of rainfall amount and δ^18^O_p_) using the technique described below (see ‘YOK-G TC Reconstruction’). A comparison with the San Salvador GNIP station data over the period 1968 to 1983 confirms that annually interpolated YOK-G δ^18^O values are positively correlated with mean hurricane season δ^18^O (r^2^ = 0.41, *p* = 0.01) and negatively correlated with both YOK-G_TC_ count (r^2^ = 0.41, *p* = 0.01) and HURDAT2 western Caribbean TC count (r^2^ = 0.47, *p* < 0.01), strongly supporting our interpretation of YOK-G δ^18^O as partially reflecting rainfall isotope ratio (see Supplementary Information).

### HURDAT2 Data

An instrumental/documentary record of TC activity is available back to 1850 A.D. in the form of the revised Atlantic Hurricane Database (HURDAT2)^20^,^56^. The database was filtered to include only tropical storms (TS) and hurricanes (Hu) whose tracks passed west of 75°W longitude within the Caribbean Sea (between 8 and 22°N latitude and between 61 and 89° W longitude). The resulting western Caribbean TC count was then used to calibrate the YOK-G isotope composite record (Fig. 1) as described below.

### YOK-G_TC_ Reconstruction

To build the reconstruction, the monthly-scale YOK-G δ^13^C and δ^18^O datasets were first converted to annual-scale using a 12-month moving average (MA) filter and sampling the resulting sequences at the start, middle, or the end of the year to identify the best fit. To test the hypothesis that HURDAT2 western Caribbean TC number is linearly correlated with δ^18^O and δ^13^C values, the isotope ratios were considered both individually and together, generating nine different models in addition to the ‘null model’ of no dependence. The model parameters were chosen according to the best fit coefficients identified by maximizing the log-likelihood (LL) based on a Poisson distribution. A Poisson distribution was favoured over least means squares due to the small mean of the annual HURDAT2 western Caribbean TC count. To test the significance robustly a bootstrap method was used. One million random permutations of the data were generated and the same fitting procedure was used to generate a distribution of LL values (Fig. 1c). The ideal model (λ), significant at the 99.8% level, was determined to be:


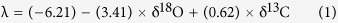


where λ is the annual YOK-G_TC_ count and δ^18^O and δ^13^C (measured in ‰ VPDB) are the annually interpolated (sampled at the middle of the year) oxygen and carbon isotope data, respectively. The correlation between the YOK-G_TC_ reconstruction and the annual HURDAT2 western Caribbean TC count (Fig. 1) is stronger for the interval 1900–1983, when the HURDAT2 dataset is more reliable (i.e., minimal undercount bias exists), but the fit parameters (−1.36, −1.92, 0.49) and significance (99.7%) are not considerably different if the HURDAT2 range 1870–1983 is used. Our bootstrap approach is robust to overfitting errors, and therefore does not require a cross-validation approach. Although stalagmite stable isotope data are typically auto-correlated due to their formation mechanism (i.e., the storage component of karst groundwater integrates rainwater on the scale of days to months), the TC count is not (<0.1). Therefore, more complicated models such as an auto-regressive moving average (ARMAX) model were not considered necessary.

## Additional Information

**How to cite this article**: Baldini, L. M. *et al*. Persistent northward North Atlantic tropical cyclone track migration over the past five centuries. *Sci. Rep.* **6**, 37522; doi: 10.1038/srep37522 (2016).

**Publisher's note:** Springer Nature remains neutral with regard to jurisdictional claims in published maps and institutional affiliations.

## Supplementary Material

Supplementary Information
