# Supplementary material for: Persistent northward North Atlantic tropical cyclone track migration over the past five centuries
Source: Sci Rep. 2016 Nov 23;6:37522. doi: 10.1038/srep37522 (PMC5120344; doi:10.1038/srep37522)
Supplement: Supplementary Information [file srep37522-s1.pdf]

SUPPLEMENTARY INFORMATION

# Persistent northward North Atlantic tropical cyclone track migration over the past five centuries

Lisa M. Baldini[1*#], James U.L. Baldini[1], Jim McElwaine[1], Amy Benoit Frappier[2], Yemane Asmerom[3], Kam-biu Liu[4], Keith Prufer[5], Harriet E. Ridley[1], Victor Polyak[3], Douglas J. Kennett[6], Colin G. Macpherson[1], Valorie V. Aquino[5], Jaime Awe[7,8] and Sebastian F. M. Breitenbach[9,10]

[1]Department of Earth Sciences, Durham University, Durham, DH1 3LE, UK

[2]Department of Geosciences, Skidmore College, 815 North Broadway, Saratoga Springs, New York, 12866, USA

[3]Department of Earth and Planetary Sciences, University of New Mexico, Albuquerque, NM 87106, USA

[4]Department of Oceanography and Coastal Sciences, Louisiana State University, Baton Rouge, LA 70803, USA

[5]Department of Anthropology, University of New Mexico, Albuquerque, NM 87106, USA

[6]Department of Anthropology, The Pennsylvania State University, University Park, Pennsylvania 16802, USA

[7]Institute of Archaeology, Belmopan, Belize

[8]Department of Anthropology, Northern Arizona University, Flagstaff, AZ 86011-5200, USA

[9]Department of Earth Sciences, University of Cambridge, Downing Street, Cambridge, CB2 3EQ, UK

[10]Institute for Geology, Mineralogy & Geophysics, Ruhr-Universität Bochum, Unversitätsstr. 150, 44801, Bochum, Germany

*Correspondence to: l.m.baldini@durham.ac.uk
[#]Current address: Department of Geography, Durham University, Durham, DH1 3LE, UK

**Detecting western Caribbean TC activity in regional precipitation and YOK-G $\delta^{18}O$**

Within Central America and the Caribbean, only nine Global Network of Isotopes in Precipitation (GNIP) stations have continuous datasets longer than three years. Of these, San Salvador (SS) (El Salvador; 13° 42' 0" N, 89° 7' 12" W) has the most complete precipitation $\delta^{18}O$ ($\delta^{18}O_p$) dataset (98 monthly $\delta^{18}O$ values from 1968 to 1984) and is sufficiently close (within 280 km) to Yok Balum to potentially represent regional $\delta^{18}O_p$. Large-scale similarities in rainfall amount between SS and Punta Gorda (PG) (i.e., the closest meteorological station to Yok Balum) are driven by regional ocean temperatures, ITCZ position, trade wind strength, ENSO, and tropical waves; consequently, SS GNIP rainfall amount data and local PG rainfall amount are strongly positively correlated at the monthly scale ($r^2 = 0.82$, $p < 0.001$). Mean monthly SS $\delta^{18}O_p$ is also significantly negatively correlated with mean monthly PG rainfall amount ($r^2 = 0.42$, $p < 0.01$), and ISOSCAPE modelling shows that SS and PG annual stable isotope ratios in the rainfall are also highly correlated (http://wateriso.utah.edu/waterisotopes/pages/data_access/figures.html[64]. For the months March to December (for which SS $\delta^{18}O_p$ data are available (no data during peak dry season)), mean monthly SS $\delta^{18}O_p$ is significantly negatively correlated with mean monthly SS rainfall amount ($r^2 = 0.62$, $p < 0.01$) and exhibits no correlation with mean monthly SS temperature ($r^2 = 0.03$, $p = 0.6$). In agreement with previous research[64,65], this analysis strongly suggests that rainfall amount is the dominant control on $\delta^{18}O_p$ regionally, and that SS $\delta^{18}O_p$ is broadly representative of $\delta^{18}O_p$ at our site.

The isotopically anomalous, heavy rainfall that is associated with TCs can generate strong $\delta^{18}O$ signals in speleothems on weekly to interannual timescales[66,67]. To investigate the extent that annual YOK-G $\delta^{18}O$ values are likely to reflect the isotope composition of regional TC-influenced rainfall, these data were compared to mean SS hurricane season (Aug-Oct) $\delta^{18}O_p$ data (as a proxy for PG hurricane season $\delta^{18}O_p$). For the period 1968 to 1983, YOK-G $\delta^{18}O$ values are positively correlated with mean SS hurricane season $\delta^{18}O_p$ ($r^2 = 0.41$, $p = 0.01$) and negatively correlated with both YOK-$G_{TC}$ count ($r^2 = 0.41$, $p = 0.01$) and HURDAT2 western Caribbean TC count ($r^2 = 0.47$, $p < 0.01$) (Supplementary Fig. 1), very strongly supporting our analysis. During the hurricane season, the YOK-$G_{TC}$ count therefore captures the clear association between regional TC activity and the isotopic composition of monthly rainfall across the western Caribbean. This regional-scale TC signal is similar to that exploited by previous TC rainfall proxy-based palaeotempestology studies[68-73] that use a storm signal capture radius on the order of a few 100 km (approximating the size of a typical TC rain shield[74]). Observational and modelling studies demonstrate that a strongly negative TC $\delta^{18}O_p$ signal can extend for several 100 km from the eye of the storm[48-52,56,57]. Based on this analysis of modern GNIP and meteorological station data, we interpret stalagmite YOK-G $\delta^{18}O$ as partially reflecting rainfall from TC events passing within a few 100 km of the cave site, and that these events are representative of TC activity in the broader western Caribbean region.

**Integrating North Atlantic documentary and proxy TC datasets (Fig. 3)**

Previously published historical datasets for Bermuda, Florida, Puerto Rico, and Jamaica were compiled from various documentary data sources in previous studies[75,76]. As with any historical dataset, limitations to the completeness of the records (i.e., missing data are possible and likely) relative to the modern instrumental record exist. However, these records are among the most authoritative historical TC datasets available. Although some imprecision surrounding the exact hurricane counts per 50-year time interval exists[75], the overall trends are generally considered reliable. To facilitate inter-site comparison, all documentary and proxy TC counts per 50-year time slice were normalized relative to the full length of each dataset (not extending earlier than 1550 in the case of the basin-wide dataset presented in Fig. 3a). Because the YOK-$G_{TC}$ record only extends to 1983, the final 50-year time slice in Fig. 3f was calculated using the HURDAT2 western Caribbean TC count.

Previously published documentary information spanning the period 1551 to 1998[75,76] suggests that the relative % of hurricanes affecting Bermuda, Florida, Puerto Rico, and Jamaica has decreased since 1950 (Fig. 3b-e), however, this downward trend is not reflected in the relative % basin-wide TC occurrence calculated by Mann et al.[77] (Fig. 3a). We used NOAA's historical hurricane track tool (https://coast.noaa.gov/hurricanes/) to investigate whether this discrepancy is the result of i) the minor truncation of the last 50 year time slice of the historical data (the last time slice only extends to 1998 rather than 2000) or ii) there is an undercount in the documentary data due to some TCs missing the sites. TCs (including all tropical storms and hurricanes) that passed within a 320 km

radius of each site (centered on the longitude and latitude provided in Fig. 3) were identified and grouped into 10 year time slices. A search radius of 320 km was chosen to increase the likelihood that additional TCs not recorded in the documentary datasets would be detected and because $^{18}O$-depleted TC rainfall can extend several 100 km from the eye of the storm[78-84]. Once TCs were identified, the same technique used to construct Figure 3 was followed but 10 year bins instead of 50 year bins were selected to optimize data resolution. The % TC occurrence relative to each site's full 1850 to 2000 dataset was then determined (Supplementary Fig. 2). This analysis using the more comprehensive historical hurricane tracks tool provides greater detail since 1900 A.D. than the previously published 50 year time slice data[75,76] and confirms the general upward trend in basin-wide activity observed in Figure 3a. However, the increase is not evenly distributed across the basin; Puerto Rico and Jamaica have experienced decreased TC numbers over the last 50 years, whereas more TCs passed within 320 km of Bermuda and Florida. This analysis suggests that many TCs are passing between Bermuda and Florida, as well as moving into the open Atlantic to the east of Bermuda. The basin-wide TC number is in fact greater from 1950-2000, but this increase is not evenly distributed; activity decreased at the southwestern sites but increased at the northwestern sites, consistent with the longer term conclusions of this study.

**The YOK-$G_{TC}$ count compared to regional TC reconstructions**

Several TC reconstructions of varying chronological precision and sampling resolution exist from the western Caribbean[85-87]. Perhaps the most relevant to the present study is that of Denommee et al.[85] based on a marine sediment core from Lighthouse Reef, off the

coast of northern Belize (Supplementary Fig. 3a). The Denommee et al. TC reconstruction[85] includes both hurricanes and tropical storms, but the lower resolution of their 20-year bins complicates direct comparison with the annual TC variability present in the YOK-$G_{TC}$ count (Supplementary Fig. 3a). Despite this, the low frequency trends in TC activity are reproduced in both records, particularly the peak in western Caribbean TC activity at 1650 followed by a decreasing trend until 1870 (Supplementary Fig. 3). Post-1870, the Denommee reconstruction suggests a greater number of TCs impacting Belize than the YOK-$G_{TC}$ count, peaking at ~1940 (Supplementary Fig. 3a). A second marine-based study from Belize[88] also tentatively concluded that Belizean TC activity increased after 1800. The post-1870 discrepancy between our record (strongly suggesting decreasing TC landfalls since 1800) and this marine-based TC reconstruction may reflect the preferential preservation of more intense storms (Category 3 and above) in the marine reconstructions while the YOK-$G_{TC}$ reconstruction preserves storms with low $\delta^{18}O_p$ and high rainfall volume regardless of category (which is based on wind strength). We suggest that these two results are complementary, and, when considered together, suggest that although TC activity in the western Caribbean has decreased gradually since 1650, the intensity of storms impacting Belize may have increased since 1870.

Overwash deposits from Belize have been used to reconstruct TC activity in Belize extending back several millennia[86,89]. These generally low-resolution studies suggest enhanced Belizean TC activity between 1350 and 1750, consistent with the peak observed in the YOK-$G_{TC}$ count during the LIA. These Belizean records, when compared to counterparts across the North Atlantic TC basin, also suggest the existence of an

inverse relationship between TC activity in different regions of the North Atlantic, supporting our conclusions.

Malaizé et al.[90] used lacustrine sediments deposits from the eastern Caribbean to reconstruct hurricane activity over the past 3500 years. Unfortunately, this record is very intermittent over the past 400 years preventing direct comparison with the YOK-$G_{TC}$ record. However, an out-of-phase relationship was observed between hurricanes impacting Saint-Martin and Belize and those making landfall in Puerto Rico and along the North American Atlantic Coast. Malaizé et al.[90] ascribe this out-of-phase relationship to latitudinal migration of the ITCZ consistent with the results of the present study, as well as the results other recent research[87,91].

A TC reconstruction based on the third principal component of Jamaican lake level data, termed the Extended Hurricane Activity index[92], agrees remarkably well with the YOK-$G_{TC}$ reconstruction when a 30-50 year lag adjustment is applied to the Jamaican record. Despite the slight chronological mismatch, both records exhibit a similar peak in Caribbean TC activity during the LIA (Supplementary Fig. 3b).

Overall, the low TC activity observed at the start of the YOK-$G_{TC}$ reconstruction is corroborated by several lower resolution records from the Gulf of Mexico and Caribbean[85,93-95]. Many of the differences existing between different records likely reflect chronological uncertainty or the preservation of different aspects of the tropical cyclone signal, such as wind strength versus rainfall amount. These records provide important

complementary information that permits the creation of a considerably clearer picture of how TC activity (encompassing both TC strength and frequency) changed in the past.

Records away from the western Caribbean support our interpretations. A palaeohurricane record from the NE Gulf of Mexico (GOM) is anticorrelated with the YOK-$G_{TC}$ record, consistent with our conclusions (Supplementary Fig. 3c)[94]. Overwash deposits from Puerto Rico[93] in the northeastern Caribbean reveal an increase in Caribbean TC activity during the latter half of the LIA (between 1750 and 1800). The ~100 year offset in the LIA TC activity peak between Puerto Rico and Belize (this study) is consistent with the pattern suggested here and further supports a gradual migration of dominant storm tracks from the western Caribbean to the northeastern Caribbean to the North American east coast paralleling gradual NH warming since the peak LIA. Nyberg et al.[96] reconstructed major hurricane activity back to 1730 using SST data and corals from the northeastern Caribbean (near Puerto Rico and the Dominican Republic) and marine sediment core data from the Cariaco Basin as proxies of vertical wind shear. The Nyberg reconstruction from the northern Caribbean, although only representative of major hurricane activity, exhibits remarkable agreement (out-of-phase) with the YOK-$G_{TC}$ record prior to ~1870 (Supplementary Fig. 3e). After 1870, decadal variability of the Nyberg reconstruction is strongly in-phase with YOK-$G_{TC}$ count until ~1950 when the Puerto Rican record exhibits a steep decline in TC activity that is not reflected in the YOK-$G_{TC}$ count.

**North Atlantic Oscillation (NAO) reconstructions support the YOK-$G_{TC}$ reconstruction**

Although the AMO is an important driver of North Atlantic TC frequency, BH size and position during boreal summer (i.e., the summer NAO) control the degree of Cape Verde TC track recurvature[76,97,98]. However, recent studies highlight the intrinsic variability of the NAO on short timescales (seasonal to decadal)[99] and considerable discordance exists between proxy-based NAO Index reconstructions[100]. Furthermore, a recent study of the instrumental record suggests that although the May-June NAO (MJ NAO) exerts the strongest influence on North Atlantic TC activity, the significance of this relationship is restricted to AMO cool phases[101]. A comparison of YOK-$G_{TC}$ count to the Luterbacher MJ NAO Index[102] reveals that since 1870, a negative correlation exists ($r = -0.32$; $p < 0.001$) consistent with earlier work on the TC-summer NAO relationship[76], but that this negative correlation persists regardless of AMO phase (Supplementary Fig. 4). Prior to 1870 no significant relationship exists between the YOK-$G_{TC}$ count and the MJ NAO Index, which may reflect reduced prediction skill of the models included in the MJ NAO reconstruction over this older time interval[102]. Many longer-term NAO reconstructions produce contradictory results, and longer reconstructions such as Baker et al.[103] and Ortega et al.[104] reflect predominantly winter NAO rather than the MJ NAO, complicating direct comparison to any TC reconstruction. Future research should attempt to reconstruct the NAO for the months most responsible for controlling mean TC track, and investigate whether the MJ NAO control on TC track found here back to 1870 extends further back in time.

**References**


64 Bowen, G. J. & Revenaugh, J. Interpolating the isotopic composition of modern meteoric precipitation. *Water Resour. Res.* **39**, 1299, doi: 1210.1029/2003WR002086 (2003).

65 Lachniet, M. S. & Patterson, W. P. Oxygen isotope values of precipitation and surface waters in northern Central America (Belize and Guatemala) are dominated by temperature and amount effects. *Earth Planet. Sci. Lett.* **284**, 435-446 (2009).

66 Frappier, A. B. Masking of interannual climate proxy signals by residual tropical cyclone rainwater: Evidence and challenges for low-latitude speleothem paleoclimatology. *Geochem. Geophys. Geosyst.* **14**, 3632-3647 (2013).

67 Frappier, A. B., Sahagian, D., Carpenter, S. J., González, L. A. & Frappier, B. R. Stalagmite stable isotope record of recent tropical cyclone events. *Geology* **35**, 111-114 (2007).

68 Nott, J., Haig, J., Neil, H. & Gillieson, D. Greater frequency variability of landfalling tropical cyclones at centennial compared to seasonal and decadal scales. *Earth Planet. Sci. Lett.* **255**, 367-372 (2007).

69 Miller, D. L. *et al.* Tree-ring isotope records of tropical cyclone activity. *Proc. Natl. Acad. Sci. U. S. A.* **103**, 14294-14297 (2006).

70 Frappier, A. B. *et al.* Two millennia of tropical cyclone-induced mud layers in a northern Yucatan stalagmite reveal: Multiple overlapping climatic hazards during the Maya Terminal Classic "megadroughts". *Geophys. Res. Lett.* **41**, 5148-5157 (2014).

71 Nott, J. Tropical cyclones, global climate change and the role of Quaternary studies. *J. Quat. Sci.* **26**, 468-473 (2011).

72 Haigh, I. D. *et al.* Estimating present day extreme water level exceedance probabilities around the coastline of Australia: tropical cyclone-induced storm surges. *Clim. Dyn.* **42**, 139-157 (2014).

73 Denniston, R. F. *et al.* Extreme rainfall activity in the Australian tropics reflects changes in the El NiNo/Southern Oscillation over the last two millennia. *Proc. Natl. Acad. Sci. U. S. A.* **112**, 4576-4581 (2015).

74 Matyas, C. J. Associations between the size of hurricane rain fields at landfall and their surrounding environments. *Meteorol. Atmos. Phys.* **106**, 135-148 (2010).

75 Elsner, J. B. & Kara, A. B. *Hurricanes of the North Atlantic: Climate and Society* 488 (Oxford University Press, 1999).

76 Elsner, J. B., Liu, K. B. & Kocher, B. Spatial variations in major U.S. hurricane activity: Statistics and a physical mechanism. *J. Clim.* **13**, 2293-2305 (2000).

77 Mann, M. E., Woodruff, J. D., Donnelly, J. P. & Zhang, Z. Atlantic hurricanes and climate over the past 1,500 years. *Nature* **460**, 880-883 (2009).

78 Lawrence, J. R., Gedzelman, S. D., Zhang, X. P. & Arnold, R. Stable isotope ratios of rain and vapor in 1995 hurricanes. *J. Geophys. Res.-Atmos.* **103**, 11381-11400 (1998).

79 Lawrence, J. R. Isotopic spikes from tropical cyclones in surface waters: Opportunities in hydrology and paleoclimatology. *Chem. Geol.* **144**, 153-160 (1998).

80 Lawrence, J. R. & Gedzelman, S. D. Low stable isotope ratios of tropical cyclone rains. *Geophys. Res. Lett.* **23**, 527-530 (1996).
81 Lawrence, J. R., Gedzelman, S. D., Gamache, J. & Black, M. Stable Isotope Ratios: Hurricane Olivia. *J. Atmos. Chem.* **41**, 67-82 (2002).
82 Gedzelman, S. *et al.* Probing hurricanes with stable isotopes of rain and water vapor. *Mon. Weather Rev.* **131**, 1112-1127 (2003).
83 Good, S. P., Mallia, D. V., Lin, J. C. & Bowen, G. J. Stable Isotope Analysis of Precipitation Samples Obtained via Crowdsourcing Reveals the Spatiotemporal Evolution of Superstorm Sandy. *PLoS ONE* **9**, e91117 (2014).
84 Munksgaard, N. C. *et al.* Stable Isotope Anatomy of Tropical Cyclone Ita, North-Eastern Australia, April 2014. *PLoS One* **10** (2015).
85 Denommee, K. C., Bentley, S. J. & Droxler, A. W. Climatic controls on hurricane patterns: a 1200-y near-annual record from Lighthouse Reef, Belize. *Sci. Rep.* **4**, 3876 (2014).
86 McCloskey, T. A. & Liu, K. B. A 7000 year record of paleohurricane activity from a coastal wetland in Belize. *The Holocene* **23**, 278 (2012).
87 McCloskey, T. A. & Liu, K. B. A sedimentary-based history of hurricane strikes on the southern Caribbean coast of Nicaragua. *Quat. Res.* **78**, 454-464 (2012).
88 Gischler, E., Shinn, E. A., Oschmann, W., Fiebig, J. & A., B. N. A 1500-year Holocene Caribbean climate archive from the Blue Hole, Lighthouse Reef, Belize. *J. Coast. Res.* **246**, 1495-1505 (2008).
89 McCloskey, T. A. & Keller, G. 5000 year sedimentary record of hurricane strikes on the central coast of Belize. *Quat. Int.* **195**, 53-68 (2009).
90 Malaizé, B. *et al.* Hurricanes and climate in the Caribbean during the past 3700 years BP. *The Holocene* **21**, 911-924 (2011).
91 McCloskey, T. A. & Knowles, J. T. Migration of the tropical cyclone zone throughout the Holocene in *Hurricanes and Climate Change.* (Springer Science + Business Media, LLC, 2009).
92 Burn, M. J. & Palmer, S. E. Atlantic hurricane activity during the last millennium. *Sci. Rep.* **5**, 12838 (2015).
93 Donnelly, J. P. & Woodruff, J. D. Intense hurricane activity over the past 5,000 years controlled by El Niño and the West African monsoon. *Nature* **447**, 465-468 (2007).
94 Lane, P., Donnelly, J. P., Woodruff, J. D. & Hawkes, A. D. A decadally-resolved paleohurricane record archived in the late Holocene sediments of a Florida sinkhole. *Mar. Geol.* **287**, 14-30 (2011).
95 Brandon, C. M., Woodruff, J. D., Lane, P. D. & Donnelly, J. P. Tropical cyclone wind speed constraints from resultant storm surge deposition: A 2500 year reconstruction of hurricane activity from St. Marks, Fl. *Geochem. Geophys. Geosyst.* **14** (2013).
96 Nyberg, J. *et al.* Low Atlantic hurricane activity in the 1970s and 1980s compared to the past 270 years. *Nature* **447**, 698-701 (2007).
97 Elsner, J. B. Tracking hurricanes. *Bull. Amer. Meteorol. Soc.* **84**, 353-356 (2003).
98 McCloskey, T. A., Bianchette, T. A. & Liu, K. B. Track patterns of landfalling and coastal tropical cyclones in the Atlantic basin, their relationship with the

North Atlantic Oscillation (NAO), and the potential effect of global warming. *Am. J. Clim. Chang.* **2**, 12-22 (2013).
99 Hanna, E., Cropper, T. E., Jones, P. D., Scaife, A. A. & Allan, R. Recent seasonal asymmetric changes in the NAO (a marked summer decline and increased winter variability) and associated changes in the AO and Greenland Blocking Index. *Int. J. Climatol.* **35**, 2540-2554 (2015).
100 Trouet, V., Scourse, J. D. & Raible, C. C. North Atlantic storminess and Atlantic Meridional Overturning Circulation during the last Millennium: Reconciling contradictory proxy records of NAO variability. *Glob. Planet. Chang.* **84-85**, 48-55 (2012).
101 Caron, L.-P., Boudreault, M. & Bruyére, C. L. Changes in large-scale controls of Atlantic tropical cyclone activity with the phases of the Atlantic multidecadal oscillation. *Clim. Dyn.* **44**, 1801-1821 (2015).
102 Luterbacher, J. *et al.* Extending the North Atlantic Oscillation reconstructions back to 1500. *Atmos. Sci. Lett.* **2**, 114-124 (2002).
103 Baker, A., C. Hellstrom, J., Kelly, B. F. J., Mariethoz, G. & Trouet, V. A composite annual-resolution stalagmite record of North Atlantic climate over the last three millennia. *Sci. Rep.* **5**, 10307 (2015).
104 Ortega, P. *et al.* A model-tested North Atlantic Oscillation reconstruction for the past millennium. *Nature* **523**, 71-74 (2015).

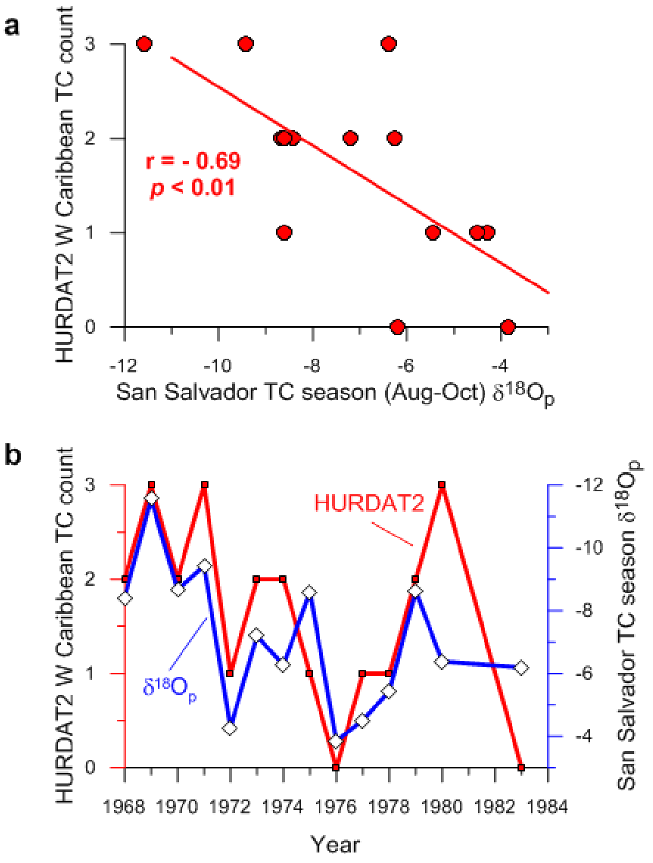


**Supplementary Figure 1.** San Salvador TC season (Aug-Oct) $\delta^{18}O_p$ for the years 1968 to 1983 (excluding 1981-1982 for which rainwater isotope data were unavailable) compared to the HURDAT2 western Caribbean TC count for the same years presented as a crossplot (**a**) and time series (**b**). In (b), the HURDAT2 western Caribbean TC count is presented in red (black squares) and San Salvador $\delta^{18}O_p$ is shown in blue (white diamonds).

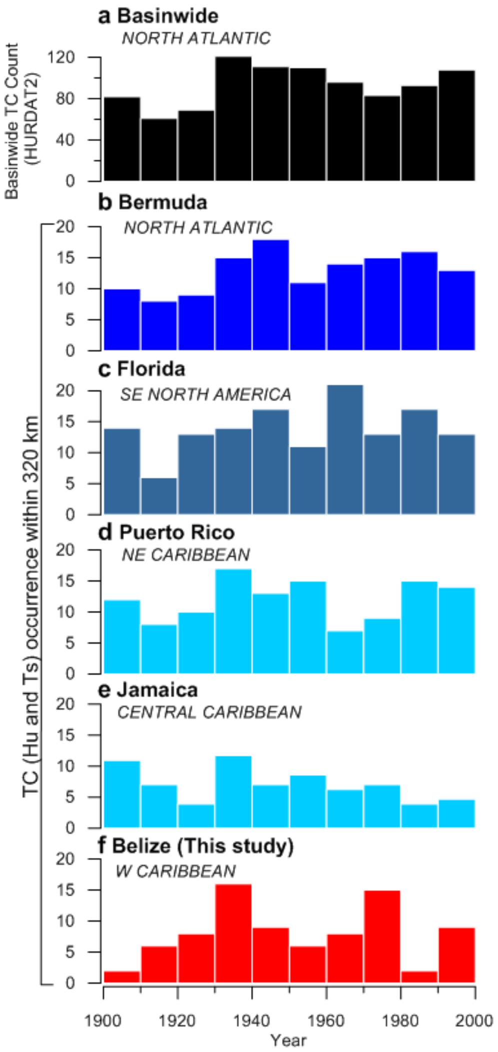


**Supplementary Figure 2.** Frequency of basin-wide TCs (**a**) and TCs that passed within a 320 km radius of locations (**b**) Bermuda, (**c**) Florida, (**d**) Puerto Rico, (**e**) Jamaica, and (**f**) Belize along the western margin of the North Atlantic. Using the NOAA Historical Hurricane Tracks tool (https://coast.noaa.gov/hurricanes/), TCs (including all tropical storms and hurricanes) that passed within 320 km of each site (centered on the longitude and latitude of each site in Fig. 3) were identified and grouped into 10 year time slices between 1900 and 2000.

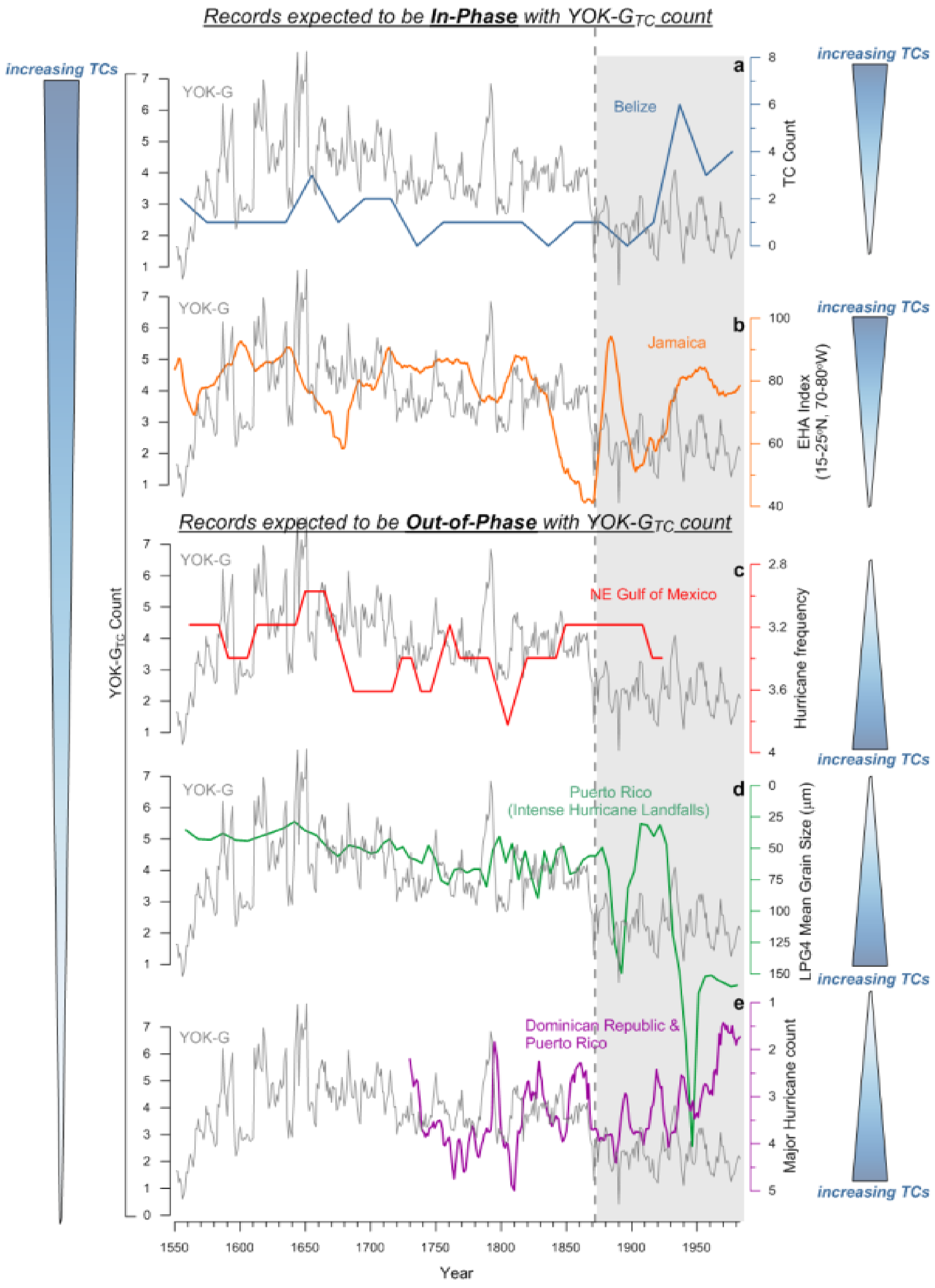
Records expected to be In-Phase with YOK-G_TC count
Records expected to be Out-of-Phase with YOK-G_TC count
increasing TCs
YOK-G
Belize
TC Count
Jamaica
EHA Index (15-25°N, 70-80°W)
NE Gulf of Mexico
Hurricane frequency
Puerto Rico (Intense Hurricane Landfalls)
LPG4 Mean Grain Size (μm)
Dominican Republic & Puerto Rico
Major Hurricane count
YOK-G_TC Count
Year
a
b
c
d
e

**Supplementary Figure 3.** The YOK-$G_{TC}$ record compared to regional TC reconstructions from (**a**) Belize[85], (**b**) Jamaica[92], (**c**) Gulf of Mexico (GOM)[94], and **d**, **e**) Puerto Rico[93,96]. Note the direction of increasing TC activity is indicated to the right and left of the axes. YOK-$G_{TC}$ count is negatively correlated with TC activity in the GOM (c) and Puerto Rico (d, e). Vertical dashed lines and grey band denote the approximate timing of the AMO polarity shift discussed in the text.

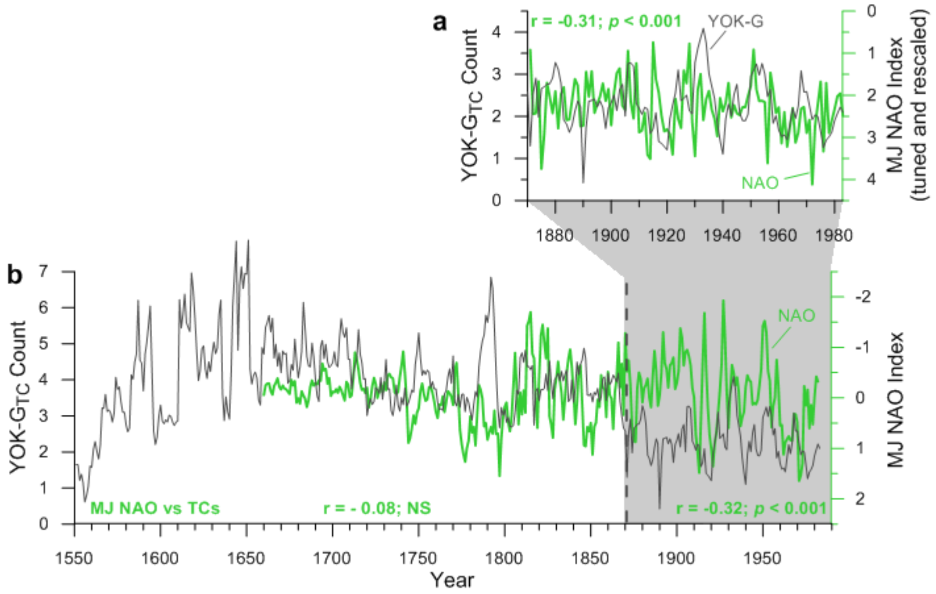


**Supplementary Figure 4.** The YOK-$G_{TC}$ reconstruction compared to a long-term reconstruction of the summer NAO. (**a**) The YOK-$G_{TC}$ count compared to the tuned (2.324 yr offset; less than chronological error of the YOK-G dataset) and rescaled (0.532 scaling factor) monthly-resolved Luterbacher NAO Index reconstruction[102] averaged over the months May and June (MJ) (green curve) over the Instrumental Era. **(b)** The full YOK-$G_{TC}$ count compared to the 3-yr moving average of the full Luterbacher MJ NAO Index reconstruction[102] (no tuning or rescaling applied). The approximate timing of the AMO polarity reversal discussed in the text is represented by a dashed grey line. The results of linear least squares regression analysis based on 5-yr moving averages of the datasets are shown.
